# Novel Pattern of Iron Deposition in the Fascicula Nigrale in Patients with Parkinson's Disease: A Pilot Study

**DOI:** 10.1155/2016/9305018

**Published:** 2016-07-04

**Authors:** Miriam E. Peckham, Khashayar Dashtipour, Barbara A. Holshouser, Camellia Kani, Alex Boscanin, Kayvan Kani, Sheri L. Harder

**Affiliations:** ^1^Department of Radiology, School of Medicine, Loma Linda University, 11234 Anderson Street, Loma Linda, CA 92354, USA; ^2^Department of Neurology, School of Medicine, Loma Linda University, 11234 Anderson Street, Loma Linda, CA 92354, USA

## Abstract

*Background and Purpose. *To determine whether the pattern of iron deposition in the fascicula nigrale in patients with Parkinson's disease would be different from age-matched controls by utilizing quantitative susceptibility mapping to measure susceptibility change.* Methods. *MRIs of the brain were obtained from 34 subjects, 18 with Parkinson's disease and 16 age- and gender-matched controls. Regions of interest were drawn around the fascicula nigrale and substantia nigra using SWI mapping software by blinded investigators. Statistical analyses were performed to determine susceptibility patterns of both of these regions.* Results. *Measurements showed significantly increased susceptibility in the substantia nigra in Parkinson's patients and an increased rostral-caudal deposition of iron in the fascicula nigrale in all subjects. This trend was exaggerated with significant correlation noted with increasing age in the Parkinson group.* Conclusion. *The pattern of an exaggerated iron deposition gradient of the fascicula nigrale in the Parkinson group could represent underlying tract dysfunction. Significant correlation of increasing iron deposition with increasing age may be a cumulative effect, possibly related to disease duration.

## 1. Introduction 

**Figure 1:**
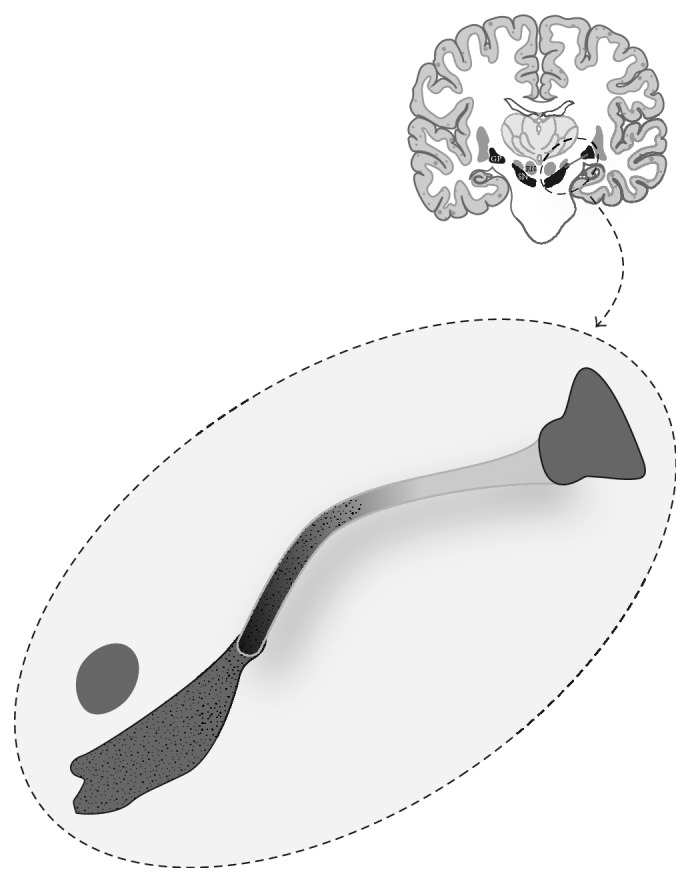
Illustration of the fascicula nigrale, a mineralized midbrain structure extending from the GP to the SN.

**Figure 2:**
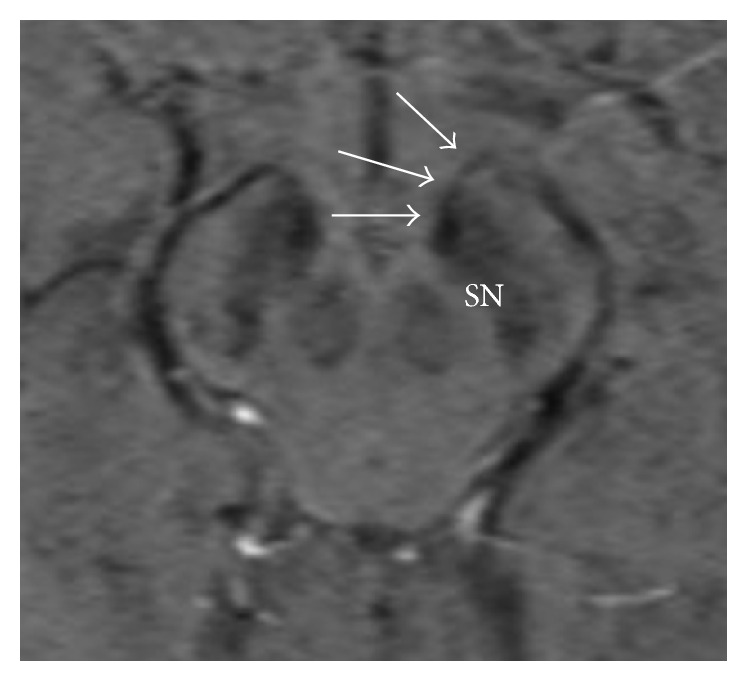
The FN (delineated by white arrows) is shown at its junction with the SN (SWI, 2 mm).

**Figure 3:**
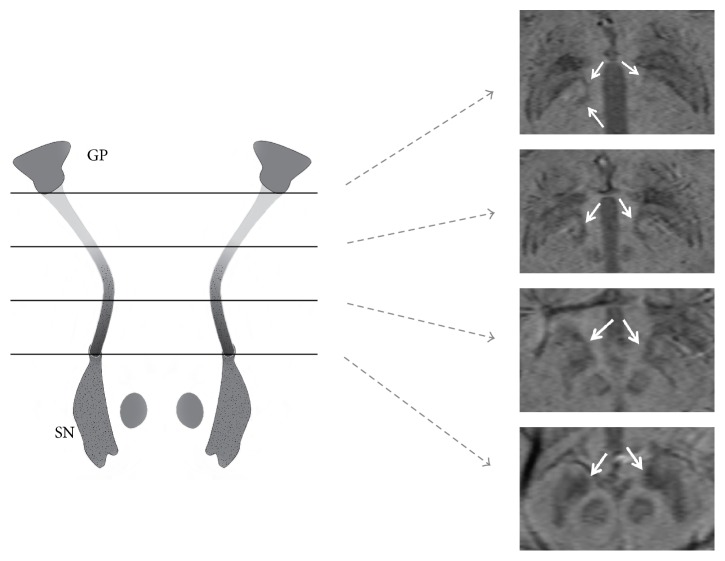
Illustration depicting the appearance of multiple slices through the FN (white arrows on SWI images, 2 mm thk) shown at its rostral junction with the globus pallidus descending to its caudal junction with the substantia nigra.

Parkinson's disease (PD) is a neurodegenerative disorder which has been found by multiple investigators to be associated with cell loss in the striatonigral dopaminergic pathway as well as the substantia nigra (SN) [1, 2]. Anatomically, the striatonigral tract and nigrostriatal tract make up the fascicula nigrale (FN) which was first described by Harder et al. as a mineralized structure extending from the globus pallidus (GP) to the substantia nigra (SN) (Figure 1) [3]. This tract has been hypothesized to take part in iron transport between the basal nuclei and midbrain [3, 4]. On susceptibility weighted imaging (SWI) this tract is identified as a linear focus of increased susceptibility extending from the medial aspect of the GP rostrally to the anterior aspect of the substantia nigra caudally (Figures 2 and 3).

The SN has been found to have increased accumulation of iron in PD by multiple investigators; because of this, the mechanism of iron homeostasis in this disease state has been a subject of interest [5, 6]. Transferrin is the brain's primary iron-transport protein and was found by Lee and Andersen to have increased levels within dopaminergic neurons of the SN in studies on rat and monkey models of PD [7]. The movement of transferrin to the storage sites has been hypothesized by Olanow and Youdim to be through axonal transport and has been studied in rats where transferrin has been tracked from the retina into the optic nerve, chiasm, and eventually the super colliculus by Moos et al. [8, 9]. Lactoferrin and DMT1 (divalent metal transporter 1), additional transporters of reactive iron, have also been found by Hirsch to be overexpressed within dopaminergic neurons of SN cells in mouse models of PD [10, 11]. Hirsch also found increased levels of DMT1 in the brain to be correlated with increasing age corresponding to known age related cellular iron accumulation as described by Hallgren and Sourander [10–12]. An increase of these transport proteins within the SN may partly account for disrupted iron homeostasis [7, 10, 11].

Hallgren and Sourander first characterized midbrain mineralization patterns in postmortem subjects in 1958 [12]. Multiple investigators have developed techniques to measure iron deposition noninvasively using high-field strength spin-echo T2 weighted MRI sequences such as relaxometry (R2*∗*), quantitative susceptibility mapping, and diffusion tensor imaging [5, 13–16]. SWI was first developed in the mid-1990s using phase as a means to enhance contrast in T2*∗* sequences. Quantitative susceptibility mapping grew out of the SWI precursor as a method to use phase information to measure iron content in the midbrain, as discussed by Haacke et al. [15]. Mapping of SWI phase shift values was positively correlated to Hallgren and Sourander's original autopsy findings in in vivo healthy controls by Zhang et al. validating the application of SWI mapping as a noninvasive technique for iron measurement [16]. Quantitative susceptibility mapping was also found to exhibit superb contrast of brain structures and substructures, some of which were not evident on R2*∗*, magnitude, or frequency images by Deistung et al. [14]. Multiple studies using noninvasive SWI and R2*∗* have been performed, replicating previous postmortem findings of increased iron deposition in the brain of patients with PD [2, 6, 17–24]. Quantitative susceptibility mapping was originally found to be more sensitive than R2*∗* in detecting iron deposition changes in patients with multiple sclerosis by Haacke et al. Murakami et al. and Barbosa et al. compared these techniques in PD brains, and quantitative susceptibility mapping was found to be more sensitive with highly accurate discrimination of patient and control groups [17, 25, 26]. As quantitative susceptibility mapping is currently the most accurate and sensitive measurement technique for midbrain iron deposition, it was used in this study to quantify iron deposition in the SN and FN.

It has been hypothesized by Graham et al. that increased susceptibility in the SN is due to accumulation of iron free radicals [5]. Iron deposition patterns in the FN have never been previously measured. Because of its involvement in the striatonigral dopaminergic pathway and its hypothesized role in iron transport, further investigation of this tract for pathologic iron deposition is warranted. Our group recently demonstrated the heterogeneity of iron accumulation in the brain, among patients with PD, using SWI [6]. The purpose of this study was to use susceptibility mapping to measure the pattern of iron deposition in the fascicula nigrale in PD patients. It was hypothesized that PD patients would have increased iron deposition in the both the FN and the SN.

## 2. Methods

### 2.1. Subjects

Eighteen patients (11 males and 7 females, mean age 69.1 years, and range: 52–82 years) with a new diagnosis of idiopathic PD were recruited by a movement disorders specialist from Loma Linda University Medical Center (LLUMC) Movement Disorder Clinic Department of Neurology, based on the UK Parkinson's Disease Society Brain Bank's criteria (Table 1). All subjects with PD had a brain MRI performed within 3 months of initial diagnoses. A PD treatment regimen was not yet implemented at the time of MRI. Patients with atypical parkinsonism, concomitant vascular parkinsonism, or parkinsonism due to neuroleptics were excluded from the study. Sixteen non-PD subjects (5 males and 11 females) with mean age of 64.4 years (range: 53–90 years) were recruited from LLUMC Neurology Clinic after ruling out PD. Subjects with past or current history of cognitive impairment, stroke, head trauma, brain lesions, or other neurodegenerative and neuropsychiatric diseases were excluded. Our Institutional Review Board approved the study. Informed consent was obtained from all study participants.

### 2.2. Magnetic Resonance Imaging and Image Processing

Conventional MR imaging and SWI were performed with a 3 T whole body MR scanner (Tim/Trio, Siemens Medical Solutions, Erlangen, Germany) using a 12-channel head array coil. The following sequences were acquired on all subjects: sagittal T1 weighted 3D MPRage (TR/TE/TI = 1950/226/900 msec, 1 × 1 × 1 mm voxel size), sagittal 3D T2 weighted SPACE, (TR/TE3200/458 msec, 1 × 1 × 1 mm voxel size), axial fat-saturated FLAIR (TR/TE/TI = 9000/77/2500 msec, 3 mm thick, .9 mm spacing), axial diffusion (EPI single shot TR/TE = 5700/103 msec, 1.5 × 1.5 × 3 mm voxel size .9 mm spacing), and axial 3D SWI (TR/TE = 29/20 msec, 1.0 × 0.5 × 2.0 mm voxel size). T1 and T2 weighted sequences were used to aid in anatomic localization.

### 2.3. Susceptibility Mapping and Region of Interest Data Analysis

**Figure 4:**
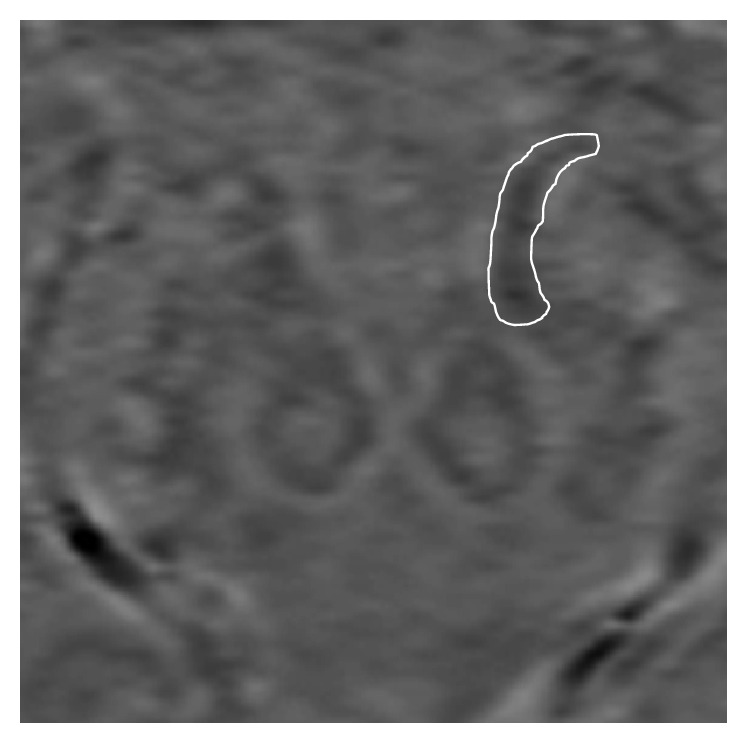
ROI around the FN as seen on mIPs of susceptibility maps (SWIM, 8 thk).

SWI mapping data were calculated with SPIN (Signal Processing In Nuclear magnetic resonance, developed by the MRI Institute for Biomedical Research, Detroit, Michigan) [27, 28]. Susceptibility maps were generated from the vertex to the medulla using magnitude and phase SWI images. The images were prefiltered by the manufacturer's software and no additional filter was applied. An inverse filter threshold of 0.1, iterative SWI mapping vein threshold of 200, *k*-space threshold of 0.1, and iterations times of 3 were applied in generating the susceptibility map. Minimum intensity maps (mIPs) were created from four slices that were each 2 mm thick. Two blinded, independent readers drew regions of interest (ROI) around the bilateral FN and SN on these mIP images for each subject. These ROIs were determined by tracing along the outside of the mineralized portion of the substantia nigra. ROI tracings were also drawn just outside of the mineralized borders of the putamen, thalami, red nuclei, dentate nuclei, and precentral gyrus (at the omega sign) at their most prominent slice bilaterally. A threshold of 1000 was applied to each ROI, obtained by averaging the subcortical white matter in all subjects (AVG 1000.06 ± 0.5). Maximum intensity values in each ROI above this threshold were recorded. The susceptibility intensity values were taken from the region of interest drawn around the SN at its most prominent slice bilaterally. The FN was measured at all slices it was visualized on from its superior connection to the globus pallidus to its inferior connection with the SN (Figure 4). The number of 8 mm slices the tract was present on ranged between 3 and 10, averaging approximately 5.0 slices (40 mm) on the right and 5.1 slices (41 mm) on the left. The FN was divided into two parts, rostral and caudal. If there were an odd number of slice measurements, then favor was given to the caudal half. In the case of a measurement agreement falling outside of the 95% confidence interval, a third reader made the final determination. A third reader had to be utilized for 4 of 68 tract measurements.

### 2.4. Statistical Analysis

All statistics analyses were performed using SPSS statistical software (version 13.0, SPSS Inc., Chicago, Illinois). *P* values less than 0.05 were considered statistically significant. Parametric tests were used to determine significant difference between the two cohorts and nonparametric tests (Mann-Whitney *U* test) were used if normality assumptions could not be met. Spearman correlation analysis was conducted to analyze the relationship between the indicators. Age and gender were modeled as covariates to correct for age- or gender-related iron deposition in the binary logistic regression analysis. To verify the reproducibility of the findings (intrarater variability) and to determine interrater reliability, intraclass correlation coefficients (ICC) were calculated.

## 3. Results 

Susceptibility intensity values for the FN were redefined by the same rater to assess the reproducibility of the results. The correlation was 0.955 comparing the rostral to caudal deltas of the FN, which is an acceptable level of reproducibility. Susceptibility intensity values of the FN were also compared between two readers showing correlation of 0.733 when comparing the rostral to caudal deltas, which is an acceptable level of reproducibility. Finally, susceptibility values of the SN were compared between two readers showing correlation of 0.871, which is an acceptable level of reproducibility.

There was significantly increased susceptibility of the SN in the PD group (*P* = 0.012).

To assess MRI detectable iron levels in the whole FN structure, the mean and standard deviation (SD) of iron deposition along the entire tract were calculated by averaging the maximum values obtained in each ROI. These results showed a trend of increased iron deposition (1083.78 versus 1069.95 in ppm of iron, *P* = 0.065) and wider standard deviation in the PD group (37.00 versus 25.77, *P* = 0.169).

**Figure 5:**
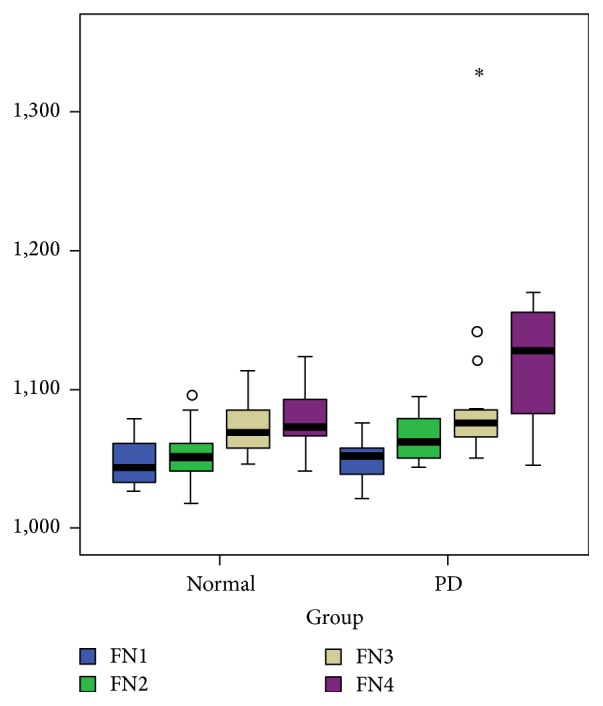
Descending pattern of increasing iron deposition along the FN (FN1 is rostral and FN4 is caudal and also contains the averages of slices more caudal to FN4 when present). *∗* = outlying value.

**Figure 6:**
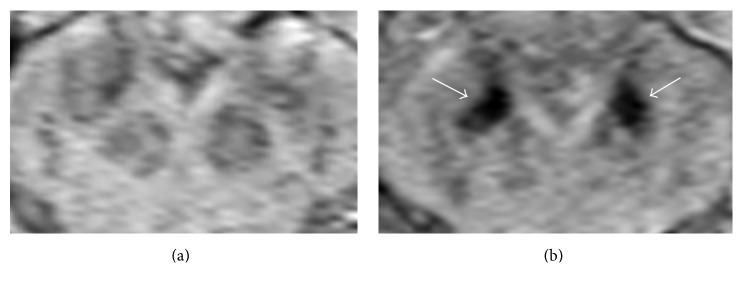
Axial images through the level of the substantia nigra demonstrating iron deposition patterns in a control subject (a) and PD subject (b), with the PD subject showing focally high susceptibility at the caudal aspect of the FN tract (white arrows) (SWI, 2 mm).

**Figure 7:**
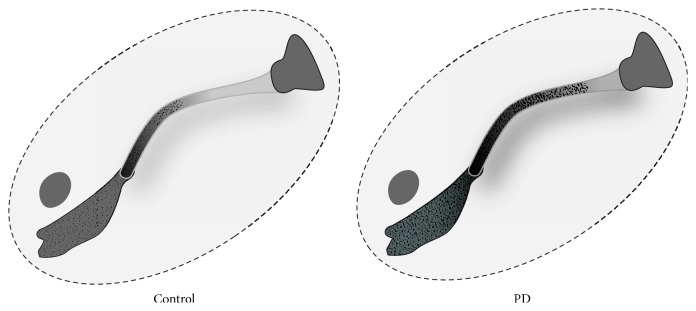
Illustrated trends of iron deposition in the FN and SN in control and PD groups.

All subjects were found to have a descending pattern of increasing iron deposition along the FN with higher susceptibility levels in the caudal aspect of the tract (Figure 5). The iron deposition at the caudal aspect of the tract showed a nonsignificant trend of increased susceptibility in the PD group compared to controls (1105.13 ± 45.8 versus 1083.00 ± 13.8 in ppm of iron, *P* = 0.073) (Figure 6). An illustrated summary of results is provided in Figure 7, representing control and PD groups, respectively.

The difference between the lowest value in the rostral and highest value in the caudal aspects of the tract (designated as delta) was calculated and showed a nearly significant trend of an increased delta in the PD group (99.9 ± 82.9 versus 57.8 ± 29.1, *P* = 0.056).

Age in the PD group was positively correlated with the mean (*r* = 0.632; *P* = 0.005) and the standard deviation of iron deposition in the FN (*r* = 0.530; *P* = 0.024) and deposition at the caudal aspect of the FN (*r* = 0.575; *P* = 0.013), as well as an increased delta (*r* = 0.626, *P* = 0.005). There was no significant correlation in the control group between age and iron deposition. Age and gender were modeled as covariates to correct for age- and sex-related iron deposition in the binary logistic regression analysis. The iron deposition at the caudal aspect of the tract, the mean, and delta was all insignificant after adjustment for age and sex.

All other measured regions of interest including the gray matter, putamen, thalami, red nuclei, and dentate nuclei showed no significant difference in iron deposition between PD and control groups.

## 4. Discussion

There is significantly increased iron deposition in the SN in the PD group, in line with multiple prior susceptibility studies [2, 5, 16, 17, 19–24]. Our results demonstrate a novel trend of exaggerated iron deposition, increasing from the rostral to caudal FN, in the PD group, with significant correlation with increased age. The findings of increased iron deposition in the FN with PD patient age and trend of an exaggerated gradient may suggest an underlying pathologic process.

The correlation of these findings with increased patient age is an interesting finding of uncertain etiology. This may be related to disease prolongation and severity, possibly while the disease was subclinical as these patients were all at early stage.

Limitations in this study include white matter contamination, which was potentially more exaggerated in the FN because of the small region of interest. Because of this maximum values were studied to decrease the effect of white matter dilution. As this was a pilot study, the number of subjects to achieve adequate power had never been previously established for reference. Some values reaching only near-significance could suggest that the overall subject size may have been too small to detect a difference between groups. Conversely, normal variability in deposition patterns across age groups as previously described by Harder et al. may have been exaggerated by the small sampling size [3]. Further studies would benefit from increased power to establish whether these deposition trends are truly abnormal in the PD group. A further limitation of the study was the uneven sex distribution between control and PD groups. To correct this discrepancy, gender and age were modeled as covariates in the binary logistic regression analysis. Another study limitation was using prefiltered data for measurement. This likely decreased the sensitivity of susceptibility measurements, which may have diluted the noted trends. A nonfiltered set of normal values would be helpful in developing a conversion factor for the filtered data.

## 5. Conclusion

PD has been associated with neuronal cell loss in the striatonigral dopaminergic pathway. The novel pattern of an exaggerated iron deposition gradient of the FN in PD could represent underlying tract dysfunction. Significant correlation of increasing iron deposition with increasing age may be a cumulative effect, related to disease duration.

## Figures and Tables

**Table 1:** Demographic and clinical characteristics of the study groups.

	PD	Control	*P* value
	*N* = 18	*N* = 16	
M : F	11 : 7	5 : 11	0.081
Mean age (years)	69.1 ± 11.2^*∗*^	64.4 ± 6.1	0.138
Time of diagnosis to MRI (months)	1.1 ± 0.3	N/A	
Handedness right : left	15 : 3	16 : 0	

Data are shown as mean ± SD.

^*∗*^Because the diagnosis was de novo, this also represents mean age of PD onset.

## Abbreviations

PD: Parkinson's disease
SN: Substantia nigra
FN: Fascicula nigrale
GP: Globus pallidus.
